# Cognition and social functioning in first episode psychosis: A systematic review of longitudinal studies

**DOI:** 10.3389/fpsyt.2023.1055012

**Published:** 2023-03-06

**Authors:** Maria José Montaner-Ferrer, Marien Gadea, Julio Sanjuán

**Affiliations:** ^1^Department of Psychobiology, Faculty of Psychology, Universitat de València, Valencia, Spain; ^2^CIBERSAM-Mental Health, Madrid, Spain; ^3^Department of Psychiatry, Faculty of Medicine, Universitat de València, Valencia, Spain

**Keywords:** first episode psychosis, social functioning, cognition, social cognition, neurocognition, longitudinal studies and systematic review

## Abstract

**Introduction:**

This systematic review aimed to answer whether we can predict subsequent social functioning in first episode psychosis (FEP) by means of an initial cognitive examination. In order to do this, we gathered longitudinal studies which evaluated neurocognition and/or social cognition regarding their impact on long-term social functioning of FEP patients.

**Methods:**

The MOOSE method was employed and 28 studies covering data from a total of 2572 patients with longitudinal trajectories from 2 months to 5 years were reviewed.

**Results:**

In general, cognitive deficits impacted on the social functioning of the FEP patients across the time. The neurocognitive domains which most closely predicted social functioning were processing speed, sustained attention and working memory. An overall cognitive dysfunction, low IQ and the academic trajectory were also found predictive. Regarding social cognition, the findings were not unanimous.

**Discussion:**

In addition of the impact of each variable, several of the articles found a complex relationship between social cognition, neurocognition, social functioning and negative symptoms, pointing social cognition as a modulator of neurocognition but being modulated as well by negative symptoms. The principal clinical implication of this review is that the initial assessment of FEP patients and their rehabilitation must take cognition into account.

## 1. Introduction

The Handbook of Social Functioning in Schizophrenia (1) defines the impairment in social functioning as the inability of individuals to meet societal defined roles such as homemaker, worker, student, spouse, family member, or friend. In addition, individual’s satisfaction with their ability to meet these roles, their ability to care for themselves and the extent of their leisure and recreational activities are often subsumed under the rubric of social functioning. It is not surprising that having problems to maintain such an active role in society can become incapacitating and undermine one’s quality of life. Despite this, social functioning in psychosis did not begin to attract the interest of researchers until the middle of the 20th century, when the psychiatry field changed its traditional paradigm of the hospitalized psychotic patient to adopted a new global approach to therapy, whose main goal was the integration of the mentally ill in the community (2). This led to broader the attention to the social impairments associated with mental disorders, and finally to the inclusion of deterioration in social and occupational functioning among the diagnostic criteria of DSM-III for schizophrenia.

Nowadays, that interest has been combined with new technological advances, resulting in the development of new antipsychotic treatments in conjunction with innovative models of community care and, more recently, new strategies for early intervention (3). These strategies have been especially useful in First Episode Psychosis (FEP), and even in individuals at Ultra High Risk of Psychosis (UHR), since intervention in the first 2 or 3 years has been shown to reduce the risk of another psychotic episode by 50%, which, in turn, reduces the possibility of making the illness worsen (4). Actually, and due to all these innovations and advances in therapeutic intervention, psychotic symptoms generally decrease in the first year after initiation of treatment in the vast majority of patients, which, hypothetically, should increase the functionality of the individual. This contrasts with the very low rates of recovery found in individuals treated for a FEP when *recovery* is defined not only in terms of psychotic episodes but, more generally, as social and clinical recovery lasting for at least 2 years (together with mild symptoms): the percentage of patients found to meet these criteria in a meta-analysis of outcome studies reach just to 13.5% (3, 5). Therefore, and with such a definition for recovery in mind, many researchers focused recently in the social functioning and the real-world adjustment of the patient (6) to determine the factors that can afford protection as well as those that represent an improved prognosis in the patient’s social life, with a large number of studies on both aspects (protection and prognosis) having been published since the beginning of this century (2). One of the main factors explored is cognition, since cognitive deficits represent one of schizophrenia’s core features, which affect patients’ functioning and recovery, and seems to be related to high levels of functional disability, even more so than psychopathology (7), what has led recently to a “white paper” of recommendations for good practitioners in therapy and rehabilitation for FEP patients, developed by experts in cognitive remediation (8).

The present review explores and systematizes longitudinal studies published in recent years regarding the above-mentioned relations in patients diagnosed with FEP. We will focus on such cognitive factors and its relation to social functioning by analyzing prospective data. We focus on studies of FEP patients because of their significance for early intervention, and also because of the need to distinguish its course from the one observed in the chronic illness, since both clinical and functional features have been shown to be different (9). Moreover, the advantage of studying this earlier phase of psychosis is that clinical components as long illness duration, aging, effects of long-term medications, chronic hospitalization and other factors related to long-term intervention do not stand as confounding factors. A systematic review found cognitive impairment across domains, up to severe level based on Cohen’s effect size, already in FEP studies (10). However, according this review, differences in levels of impairment are observed between studies, as well as within domains, indicating that further consolidation of cognitive impairment over the course of illness may be present. Thus, our aim is to review how cognitive deficits impact across the time on social functioning in patients diagnosed with FEP. The term *cognitive* is essentially broad and a complete consensus among authors categorizing cognitive domains is lacking. Therefore, given that we limited our review to FEP patients and longitudinal studies, we decided to broader the cognitive focus to include studies measuring both general aspects of cognition (like the IQ or the academical performance) as well as the domains of cognition as defined by the Neurocognitive Work Group of the DSM-5 (attention, executive function, learning and memory, language, perceptual–motor function, and the newly included social cognition domain) (11). In spite of this, and regarding the different cognitive processes, it must be noted that neurocognition has often been separated from social cognition. Neurocognition refers to the processes of linking and appraising information, and includes cognitive domains that have traditionally been referred to as “cognitive” in the literature, such as speed of processing, working memory, attention, memory, or executive functions. Social cognition refers to the mental operations underlying social interactions such as perception, interpretation, and generation of responses to the intentions, dispositions, and behaviors of others (12) and includes aspects and variables like theory of mind, social perception, social knowledge, attributional biases, and emotion processing, areas that are affected, in an heterogeneity manner, in FEP patients (13). This review will distinguish between neurocognition and social cognition as different constructs that influence each other: the exact form of their relationship is still unclear in the literature, though some studies have underlined social cognition as a mediator between neurocognition and social functioning (14), to outline their role in in FEP patients. On the other hand, only longitudinal studies have been included in the review due to their ability to outline the relation among different variables over a period of time (15). In sum, the final question this review aims to answer is whether we can predict subsequent social functioning in first episode psychosis by means of a cognitive examination. In order to do this, we have gathered information from the literature about general cognition, neurocognition, and social cognition, and we have evaluated their impact on long-term social functioning.

## 2. Methods

**Figure 1:**
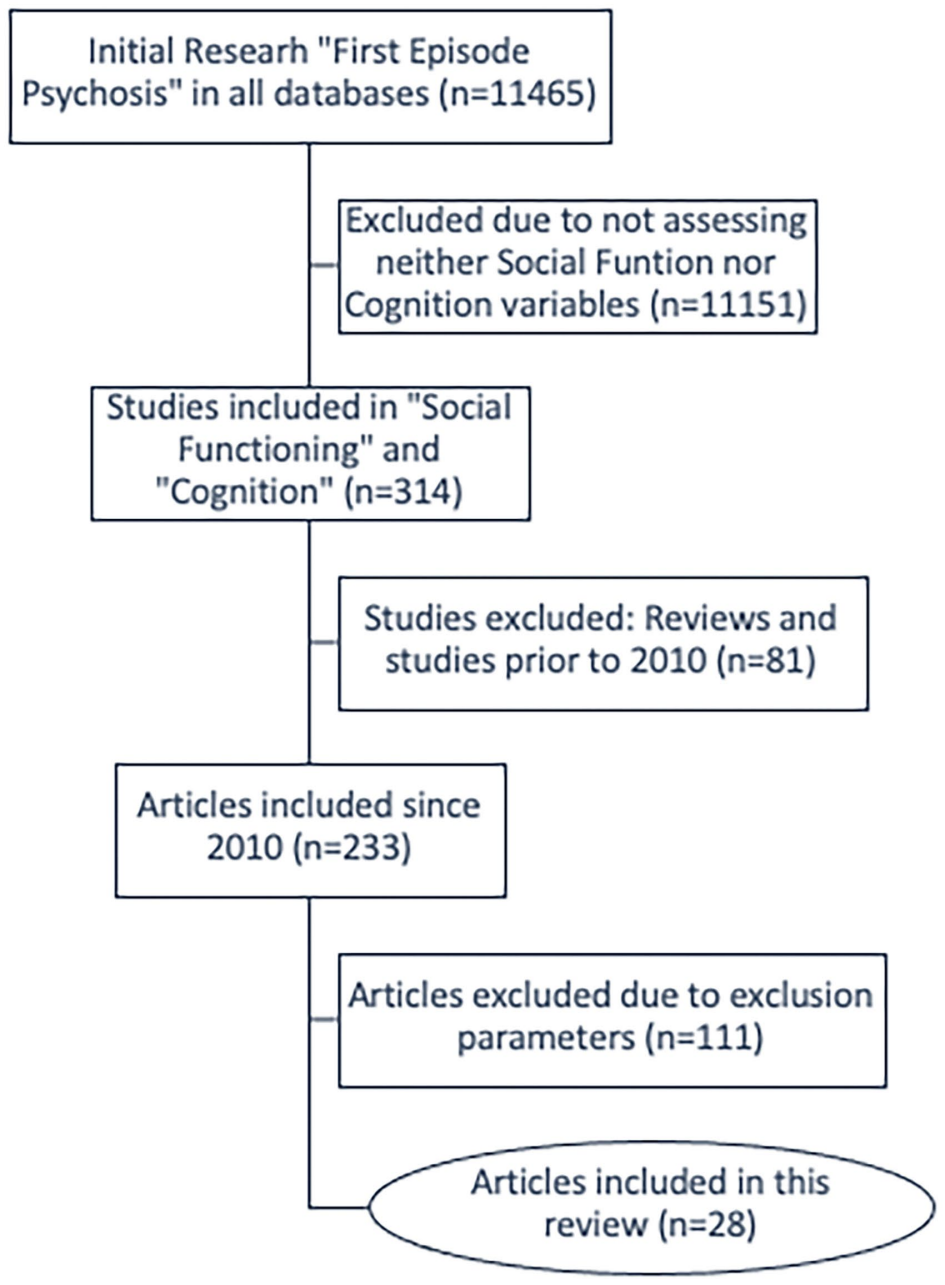
Article selection flowchart.

The MOOSE method (Meta-Analyses and Systematic Reviews of Observational Studies) was employed for this review (16). Articles of experimental studies were collected from 5 databases, all accessible within the University of Valencia: Pubmed, WoS (Web of Science), ProQuest, Dialnet and Google Scholar. The search was carried out in February 2022, and covered research carried out over an 11-years period (2010–2021). An initial search for “First Episode Psychosis” (FEP) in WoS rendered 11,465 results, which was narrowed down to 314 results when the search terms “Social Functioning” and “Cognition” were included. Eventually, having excluded reviews and older than 11-years old works, we were left with a final number of 233 articles. Our research from then on focused on title, abstract and keywords of these 233 articles in order to confirm whether or not their variables corresponded with the inclusion criteria of this review, which were the following: longitudinal studies whose variables were related to cognition and social functioning, quality of life and/or patient roles in society; which had been collected systematically and quantitatively and discussed and assessed in the conclusions of the study. The type of psychometric measures used to assess the dependent variables were social scales, such as the Social Functioning Scale (SFS), the Global Assessment of Functioning (GAF) or the Social and Occupational Functioning Assessment Scale (SOFAS), while cognition, whether social cognition or in terms of the classic domains of neurocognition, needed to have been measured using well known psychometric scales such as MATRICS, the Consensus Cognitive Battery (MCCB) or the Brief Assessment of Cognition in Schizophrenia (BACS) (17–21). The flowchart in Figure 1 reflects the decision-making process followed in the selection of the articles reviewed herein.

Eventually, 28 international experimental articles, with varied samples of adult first-episode psychotic patients of both genders were included in this review. The key information collected from these 28 articles can be consulted in Table 1, which highlights samples of participants and duration of the study, main focus, a general observation on quality of the study in statistical terms, and the results of each study regarding the social functioning of their patients. In addition, the Table 2 presents each of the clinical and cognitive scales (both for neurocognitive and social cognition variables) the reviewed studies applied in their measurements (thus, the independent variables) and also the scales they used to measure social functioning as the dependent variable. It should be noted that this table presents all the studies selected according to the aforementioned inclusion criteria, regardless of whether their results were statistically significant or not. In this way, the reader can obtain valuable information about what has been specifically studied regarding cognition and social functioning in each study.

**Table 1:** Main characteristics of the reviewed studies.

Authors	Sample and duration of the study	Focus of the study	Study quality	Results
Leeson et al. (22)	129 FEP patients in subgroups of different IQ levels and 27 controls. 3-years	IQ Trajectory and Cognitive Reserve	Covered comparability among groups. Appropriate and reproductible statistics: Schwarz’s Bayesian Criterion (BIC)	Better IQ at the beginning of the disease predicted better social functioning with regards to employment/occupation. Premorbid IQ did not
Bachman et al. (23)	35 FEP patients with adolescent onset psychosis (12-20y) and 31 controls. 1-year	Cognition and social function in Teenage Onset	Covered comparability among groups. Appropriate and reproductible statistics: Step-wise linear regression	Worse cognitive processing speed performance predicted worse social functioning
Torgalsbøen et al. (24)	28 FEP patients and 28 pairwise matched controls for age, gender and education. 6-months	Influence of neurocognition on clinical remission and real-life functioning	Covered comparability among groups. Appropriate and reproductible statistics: logistic and linear regression analyses. ROC analyses.	The ROC analyses showed attention was significantly predictive of remission. Attention/Vigilance and Working Memory at baseline significantly predicted social functioning 6 months later.
Torgalsbøen et al. (25)	28 FEP patients and 28 pairwise matched controls for age, gender and education. 2-years	Neurocognitive predictors of recovery. Rates of remission	Covered comparability among groups. Appropriate and reproductible statistics: Linear regression analyses for predictions, with Bonferroni corrections for multiple testing	Attention/vigilance and more years of education at baseline were predictors of better social and role functioning at follow-up, with 44–48% of the variance explained by the model
Uren et al. (26)	133 FEP patients and 46 controls. 6-months	To identify cognitive profiles within FEP patients, through predictive validity of cognitive clusters in relation to symptoms and functioning	Appropriate and reproductible statistics: Ward’s hierarchical agglomerative with k-means verification for the cluster analysis. Clusters were externally validated and 6-month predictive validity was also examined	Three-cluster solution according general cognition indexes based in units of SD below the means. Poorer cognitive functioning (cluster 1) was associated with poorer participation and functioning in educational, occupational, and social roles. Premorbid IQ was a significant predictor of group membership
Lindgren et al. (27)	66 FEP patients and 62 controls. 2-months	Theory of mind and social functioning. Whether ToM impairments are explained by cognitive deficits or ToM is impaired independently	Covered comparability among groups. Appropriate and reproductible statistics: ROC, including AUC and bootstrap CI. Linear regression models with adjusted R2	Level of social functioning was associated with ToM when age and gender were controlled for, and this association remained significant after controlling for general cognition
Griffiths et al. (28)	98 FEP patients and 30 controls. 1-year	Find predictors for poor global functional -GF- outcome: exploring clinical symptomatology, neurocognition, and social cognition	Covered comparability among groups. Appropriate and reproductible statistics: Separate backward logistic regressions to predict dichotomy in GF cut-off for social and role functioning. Nagelkerke R2. Exploratory mediation analyses (Sobel test)	When considered alone, better verbal memory was a significant predictor for less likelihood to have poor role functioning, and social knowledge significantly predicted social functioning. Attribution style, emotion perception and visuospatial processing were not significant. When including symptoms, the negatives were the only significant predictor for 12-month social (OR = 1.12) and role (OR = 1.16) functioning. Negative s. mediated the relationship between baseline social knowledge and social functioning, and between baseline memory and role functioning at follow-up
Pelizza et al. (29)	141 FEP (13–35y) patients and 98 non-FEP patients. 2-years	Social Cognition and its association with psychopathology and community functioning (work and social)	Appropriate and reproductible statistics: Multiple linear regression analysis	The social cognition impairment sub-score (from a pooled measure combined with a sub-score of classic cognitive domains and a sub-score of social cognition *per se*) was a significant predictor of cognitive change and depression at baseline (not tested at follow-up)
Woolverton et al. (30)	71 FEP. 6-months	Social Cognition (divided in 5 domains: attributional style, emotion recognition, social knowledge, social perception and theory of mind) and whether is associated with social functioning	Appropriate and reproductible statistics: Cross-sectional Pearson and linear regression for predictive value of SC at follow-up	There were no longitudinal associations between social cognition at baseline and any measure of social functioning at 6-month follow-up. These results held both with and without corrections for multiple comparisons
Pennou et al. (31)	37 FEP. 3-months	To evaluate the prognostic value of theory of mind, neurocognition and negative symptoms	Appropriate and reproductible statistics: multiple hierarchical linear regressions (in 3 steps and *Intro*)	The addition of the theory of mind in STEP3 of the regression model to predict independent living skills and family was associated with a significant increase in the variance explained by the model
González-Blanch et al. (32)	131 FEP in two groups: functional deficits (97) vs. functional recovery (34). 1-year	Whether global functional recovery (integrating social and occupational outcomes) after a FEP can be predicted by cognitive variables	Appropriate and reproductible statistics: Binary logistic regression with forward stepwise based on likelihood ratio, with recovery as the dependent variable	Sustained attention was a predictor of social functioning. Prediction accurate (97%) for the disabled but not for the recovered group
Popolo et al. (33)	15 FEP of age 18–30. 1-year	Cognitive assessment, social functioning and quality of life	Statistical analyses with low power to address predictions: *t*-test and Pearson correlations	Social functioning and quality of life found to be mostly independent of cognitive impairment (not correlations among variables found, except for attention and some subscales of social functioning)
Horan et al. (34)	55 FEP. 1-year	Social Cognition and functional correlates	Appropriate and reproductible statistics: 2-wave panel design (0 and 12 months) to evaluate associations using a cross-lagged panel correlational analyses	At the 12-month follow-up, better social cognition - both theory of mind and emotional processing - was related to better real-world functioning (work, independent living, and social functioning). The pattern of cross-lagged correlations suggested that the direction of causality is from baseline social cognition leading to 12-month functioning
Vestarger et al. (35)	117 FEP (18-34y). 10-months	Cognitive and clinical predictors of functionality. The associations between functional capacity and measures of real-world functioning	Linear and logistic regression analyses with backward exclusion based on Wald-test were adjusted for age, gender, and site	Verbal learning (30% variance explained), working memory (further 9%), and negative symptoms (4%) predicted 4-month functional capacity, accounting for 47% of the variance. Working memory (24%) and visual learning (6%) predicted 10-month functional capacity, accounting for a 30% of the variance
Stouten et al. (36)	153 FEP. 1-year	Psychotic symptoms and cognition as predictors of functioning	Appropriate and reproductible statistics: Backward regression models with post-hoc analyses (Holm–Bonferroni correction)	Some concrete cognitive deficits (visual learning) were stronger predictors for functionality in the areas of vocational/academic performance and social relationships problems at the follow-up. Theory of Mind was also a predictor for problems in social relationships at the follow-up
Ventura et al. (37)	48 FEP patients and 21 controls. 6-months	Theory of mind. To test whether the relation-ship between neurocognition (MATRICS) and functional outcome was mediated by ToM	Covered comparability among groups. Appropriate and reproductible statistics: Mediation analyses in consecutive model with the Sobel test	Longitudinal analysis indicated that neurocognition was a significant mediator of the significant relationship between both of the ToM variables (intentionality and appropriateness) and role functioning at 6 months
Norman et al. (38)	79 FEP. 5-years	Cognition and functional prediction. IQ	Appropriate and reproductible statistics: Hierarchical linear regression and logistic regression to assess the role of cognitive indices in predicting outcomes.	No relationship was detected between cognitive function and the social functioning score (SOFAS) at 5 years. However, robust correlations appeared between education at onset, premorbid academic adjustment, and overall IQ with full-time occupation and use of disability pension at 5 years
Faber et al. (39)	51 FEP. 2-years, with assessments at baseline, 6, 15, and 24 months	Predictive value of neurocognitive domains and clinical variables for symptomatic and functional outcome and clinical recovery	Appropriate and reproductible statistics: Binary logistic regression analysis with forward selection (likelihood ratio) was applied, with recovery as the dependent variable	Processing speed and working memory predicted recovery (recovery defined as the combination of both symptomatic and social functioning remission)
Bodén et al. (40)	46 FEP. 5-years	The predictive value of a battery of cognitive tests that assess global cognitive function, psychomotor function, processing speed, and verbal learning	Appropriate and reproductible statistics: logistic regression models with the Z-scores from the cognitive test variables as independent variables (two models: unadjusted and adjusted for antipsychotics)	Low psychomotor speed was associated with poor social functioning (OR = 3.37 adjusted for antipsychotic drug use)
Jordan et al. (41)	267 FEP (15-35). 2-years	Cognition and symptom remission in functional recovery (social and occupational functioning)	A series of stepwise regression models in consecutive blocks	Verbal memory was a significant predictor on functionality, but explained only 9% of the variance. Adding consecutive months in symptom remission explained 30%
Ayesa-Arriola et al. (42)	202 FEP patients in subgroups with (114) or without functional disability. 1 and 3-years	Cognition, premorbid social adjustment and real-world functional disability prediction	Covered comparability among groups. Appropriate and reproductible statistics: Different logistic regression models with Nagelkerke’s R2	At the end of the 3-year study, premorbid social adjustment (OR = 1.628) and years of education (OR = 1.117) were significant predictors of functional disability. Processing speed (OR = 1.575) and overall general cognitive functioning (OR = 1.665) were predictors at 1 year but not at the end of the 3-year study
Fu et al. (43)	24 FEP. 6 follow-ups over 4-years	Level of cognitive alterations and exploration of the functional trajectory of the disease. To stratify the patient group for identifying a poor outcome group early in the course of illness	Appropriate and reproductible statistics: A series of multilevel growth curve models fitted using maximum likelihood and an unstructured covariance structure	When a time × baseline interaction was tested, social functioning was significantly predicted by attention, verbal learning, and working memory. Role functioning was significantly predicted by attention, working memory, and reasoning/problem solving. Education level at baseline was significantly associated with role functioning. The other cognitive domains of the MATRICS did not significantly predict functional outcome
Matsuda et al. (44)	26 FEP. 5-years. Assessments every 6-months	Course of neurocognitive deficits. Find whether there is a generalized or specific domain of neurocognitive deficits that endure, improve, or deteriorate	Statistical analyses with low power to address predictions: t-test, ANOVA, Pearson correlations	Some cognitive domains worsen at 5 years (verbal memory and executive functions), but there was no correlation between any of them with social functioning
Peña et al. (45)	95 FEP. 2-years	Cognition and other variables as predictors of functional outcome in schizophrenia and non-schizophrenia syndromes after a FEP	Appropriate and reproductible statistics: multiple stepwise regressions (forward)	Worse visuospatial processing at baseline predicted poorer functional outcome for pooled FEP patients. Processing speed predicted most functional outcome measures in schizophrenia, whereas visuospatial functioning was the only significant predictor of functional outcomes in the non-schizophrenia group
González-Ortega et al. (46)	282 FEP. 2-years	To analyze the influence of social cognition as a mediator between cognitive reserve (IQ plus occupational attainment) and cognitive domains on functioning in FEP both at baseline and at 2 years	Appropriate and reproductible statistics: A three-steps path analysis for testing the mediating model, with linear regressions adjusted for potential confounders (stepwise and bootstrap)	In a first path, cognitive reserve and verbal memory were significantly related to functioning. A second path identified social cognition acting as a mediator between cognitive reserve and functioning, and between verbal memory and functioning, both of them at follow-up (not at baseline)
Oomen et al. (47)	204 FEP and 40 controls. 1-year	Cognitive subtypes, characterized by differences in both clinical and functional outcomes at baseline and follow-up	Appropriate and reproductible statistics: A hierarchical clustering approach (HCA) with Ward method and K-means	General functioning (GAF) in the severely impaired cluster was significantly lower than in those with preserved cognition at baseline and showed trend-level effects at 6- and 12-month follow-up
Sullivan et al. (48)	54 FEP. 1-year	To investigate the longitudinal association between both theory of mind and psychotic symptoms and social functioning outcome	Appropriate and reproductible statistics: Random effects multivariate linear regression models	Theory of mind was stable over time. Theory of mind at baseline was not correlated (therefore it was not considered a predictor) with social functioning at 6 and 12 months
Wright et al. (49)	80 FEP evaluated but only 26 followed 3-years	Whether neurocognition, functional capacity and metacognitive ability predicted functional outcome	A series of single regression exploratory analyses and a stepwise regression model in consecutive blocks controlling for small size of the sample	Metacognitive ability was a significant predictor of change in functional outcome from baseline to follow-up (72% variance explained). Negative symptoms did not change the model

**Table 2:** Psychometric scales which each reviewed study applied for the measuring of the independent (clinical and cognitive) and dependent (social functioning) variables.

Authors	Clinical, cognitive, and/or social cognition scales	Social functioning scales
Leeson et al. (22)	DIP, SAPS, SANS, Hamilton Rating Scale, the Young Mania Rating Scale. SAI, WAIS-III, WTAR, FSIQ, RAVLT and the Cambridge Automated Neuropsychological Test Battery	SFS and PAS
Bachman et al. (23)	SCID-I, FIGS, BPRS, BDI-II, WASI, the Computerized Category bTest from the Halstead-Reitan battery, WAIS-III, WMS-III, FAS, Animal Naming, TMT, the Children’s Memory Scale, CVLT-II, Purdue Pegboard test and the Halstead Finger Tapping Test	Global Functioning: Social and Role scales and GAF
Torgalsbøen et al. (24)	SCID-I, PANSS and MCCB	Global Functioning: Social (GF:Social) and the Global Functioning: Role (GF: Role)
Torgalsbøen et al. (25)	SCID-I, PANSS and MCCB	Global Functioning: Social (GF:Social) and the Global Functioning: Role (GF: Role)
Uren et al. (26)	WRAT-4, SCID I/P, SANS, BPRS, Picture Sequencing Task, SDMT, Picture Sequencing Task false belief subscale, False Belief and Deception Stories Task, the Hinting Task, DANVA, TMT, WAIS-III and RCFT	SOFAS
Lindgren et al. (27)	The Hinting Task, WAIS, WMS, TMT, BPRS-E and SCID-I.	GAF
Griffiths et al. (28)	MINI, PANSS, WMS-IV, WAIS-IV, the Picture sequencing Task, MSCEIT, AIHQ and SKQ	Global Functioning: Social (GF:Social), the Global Functioning: Role (GF: Role) and PAS
Pelizza et al. (29)	SCID-I, CAARMS and GEOPTE scale	CAARMS- SOFAS
Woolverton et al. (30)	IPSAQ, FEIT, FEDT, SFRT, SCRT and The Hinting Task	SFS
Pennou et al. (31)	BPRS-E, Digit Span (Forward and Backward), WAIS, COWAT, TMT and The Combined Stories Task	FESFS
González-Blanch et al. (32)	SAPS, SANS, the Hamilton Depression Scale, RAVLT, TMT, the Grooved Pegboard, Continuous Performance Test Degraded-Stimulus and Brief Test of Attention	DAS and PAS
Popolo et al. (33)	RAVLT, WCST, SEL/AT, Spinnler attention matrices, FAS, CPM and BPRS	CGI, HoNOS, VGF and Q-LES-Q
Horan et al. (34)	BPRS, MSCEIT and TASIT (Part III)	Relations Across Domains (RAD) and RFS
Vestarger et al. (35)	MCCB, PANSS and the Rosenberg’s Self-Esteem Scale	GAF and UPSA-B
Stouten et al. (36)	SCAN, BAI, BDI-II, PANSS, IRAOS, CPT 3–7, WAIS-III, TMT, Category Fluency Animal Naming, RAVLT, BVMT-R, ANT, Hinting Task and Davos Assessment of Cognitive Biases Scale	PSP
Ventura et al. (37)	Social Animations Task, SANS, SAPS and MCCB	RFS
Norman et al. (38)	CORS, PAS, NART-R, TMT, WAIS-III, the Continuous Performance Test, PASAT, WMS-III, WCST, the Thurstone Word Fluency Tests and a Controlled Oral Word Fluency Test	SOFAS
Faber et al. (39)	SCAN, PANSS, WHOQoL-BREF, the Stroop color naming and Stroop color-word naming tests. CPT-RT, TMT, Verbal Fluency, WAIS-III, CVLT and Fingertapping	GSDS
Bodén et al. (40)	SRB, TMT, Test de Golpeo, Claeson – Dahl Verbal Learning Test and SCI-PANSS	Strauss – Carpenter Functioning Scale
Jordan et al. (41)	SAPS, SANS, WMS-III, MATRICS, Circumstances of Onset and Relapse Schedule and PAS.	The Strauss-Carpenter Scale
Ayesa-Arriola et al. (42)	SCID-I, SANS, SAPS, RAVLT, Rey Complex Figure, TMT, WAIS-III, GP and CPT	PAS and DAS
Fu et al. (43)	SCID-I and MCCB	Global Functioning: Social (GF:Social) and the Global Functioning: Role (GF: Role
Matsuda et al. (44)	PANSS, JART and BACS-J	GAF and LSP-J
Peña et al. (45)	PANSS, Young Mania Scale, Montgomery-Asberg Depression Scale, SUMD, WAIS-III, BTA, Semantic and Phonological Fluency, WMS-III, Rey Complex Figure, TMT and Card Sorting Test	GAF, CGI and DAS-WHO
González-Ortega et al. (46)	SCID-I, FAST, EuropASI, The Hollingshead–Redlich Scale, WAIS-III, WISC-IV, TMT, Stroop Test, CPT-II, TAVEC, FAS Test and WCST	MCCB (Mayer-Salovey-Caruso Emotional Intelligence Test)
Oomen et al. (47)	PANSS, BACS	GAF, WHODAS 2.0
Sullivan et al. (48)	The Hinting Test, The Visual Cartoons test, TMT, the Logical Memory Test, Calgary Depression Scale, PANSS and NART	SOFAS
Wright et al. (49)	MAI, WMS-III, TMT, Verbal Fluency, Vocabulary and Matrix reasoning tasks and PANSS	TUS and UCSD

AIHQ, Ambiguous Intentions Hostility Questionnaire; ANT, Amsterdam Neuropsychological Tasks; BACS-J, Brief Assessment of Cognition in Schizophrenia, Japanese version; BAI, Beck Anxiety Inventory; BDI-II, Beck Depression Inventory-II; BPRS, Brief Psychiatric Rating Scale; BPRS-E, Brief Psychiatric Rating Scale — Extended version; BTA, Brief Test of Attention; BVMT-R, Brief Visuospatial Memory Task Revised; CAARMS, Comprehensive Assessment of At-Risk Mental States interview; CAARMS-SOFAS, Social and Occupational Functioning Scale integrated inside CAARMS; CGI, Clinical Global Impression; CORS, Circumstances of Onset and Relapse Schedule; COWAT, The Controlled Oral Word Association Test; CPM, The Colored Progressive Matrices; CPT-II, Conners’ Continuous Performance Test; CPT-RT, Continuous Performance Test, sustained attention version, reaction time in milliseconds; CVLT, California Verbal Learning Test; CVLT-II, California Verbal Learning Test-Second Edition; DANVA, Diagnostic Analysis of Nonverbal Accuracy-2–Adult Version; DAS, The Disability Assessment Scale; DAS-WHO, the Disability Assessment Schedule, World Health Organization; DIP, Diagnosis Interview for Psychosis; EuropAsi, the European Adaptation of a Multidimensional Assessment Instrument for Drug and Alcohol Dependence; FAS Test, Controlled Oral Word Association Test; FAST, The Functional Assessment Short Test; FEIT, The Facial Emotion Identification Test; FEDT, The Facial Emotion Discrimination Test; FEP, First Episode Psychosis; FESFS, The First Episode Social Functioning Scale; FIGS, Family Interview for Genetic Studies; FSIQ, Prorated full-scale IQ; GAF, Global Assessment of Functioning; GEOPTE scale, Spanish Group for the Optimization and Treatment of Schizophrenia Scale of Social Cognition for psychosis; GP, Grooved Pegboard Handedness; GSDS, the Groningen Social Disabilities Schedule; HoNOS, Health of the Nation Outcome Scale; IPSAQ, The Internal, Personal, and Situational Attributions Questionnaire; IRAOS, the Instrument for the Retrospective Assessment of the Onset of Schizophrenia; JART, The National Adult Reading Test, Japanese version; LSP-J, Life Skills Profile, Japanese version; MAI, Metacognitive Awareness Interview; MATRICS, Measurement and Treatment Research to Improve Cognition in Schizophrenia; MCCB, MATRICS Consensus Cognitive Battery; MINI, The Mini International Neuropsychiatric Interview; MSCEIT, Mayer-Salovey-Caruso Emotional Intelligence Test; NART, The Nelson Adult Reading Test; NART-R, National Adult Reading—Revised Test; PANSS, Positive and Negative Syndrome Scale y SCI-PANSS, Structured Clinical Interview for the Positive and Negative Syndrome Scale; PAS, Premorbid Adjustment Scale; PASAT, Paced Auditory Serial Addition Test; PSP, The Personal and Social Performance scale; Q-LES-Q, Quality of Life Enjoyment and Satisfaction Questionnaire; RAVLT, The Rey Auditory Verbal Learning Test; RCFT, Rey-Osterrieth Complex Figure Test; RFS, Role Functioning Scale; SAI, Schedule for the Assessment of Insight; SANS y SAPS, Scale for the Assessment of Negative/Positive Symptoms; SCAN, Schedules for Clinical Assessment in Neuropsychiatry; SCRT, The Social Cue Recognition Task; SCID, Structured Clinical Interview for DSM-IV; SCID-I, First Edition; SCID I/P, Patient; SDMT, Symbol Digit Modalities Test; SEL/AT, Span Selective Attention; SFRT, The Situational Feature Recognition Test; SFS, Social Functioning Scale; SKQ, The Social Knowledge Questionnaire; SOFAS, Social and Occupational Functioning Scale; SRB, Synonyms, Reasoning, and Block Design; SUMD, the Scale to assess Unawareness of Mental Disorders; TASIT, The Awareness of Social Inference Test; TAVEC, The Verbal Learning Test España-Complutense; TMT, Trail Making Test; A o B; TUS, Time-Use Survey; UCSD, Performance-Based Skills Assessment; UPSA-B, University of California, San Diego Performance Based Skill Assessment—Brief Version; VGF, Valutazione Globale Del Funzionamento; WAIS-III, Wechsler Adult Intelligence Scale, Third edition; WAIS-III, Wechsler Adult Intelligence Scale, Fourth edition; WASI, Wechsler Abbreviated Scale of Intelligence; WCST, Wisconsin Card Sorting Task; WHOQoL-BREF, World Health Organization Quality of Life scale-abbreviated version; WISC-IV, Wechsler Intelligence Scale for Children-IV; WMS, Weschler Memory Scale; WMS-III, Weschler Memory Scale, Third edition; WMS-IV, Weschler Memory Scale, Fourth edition; WRAT-4, The Wide Range Achievement Test, Fourth Edition; WTAR, Wechsler Test of Adult Reading.

## 3. Results

Longitudinal articles published between 2010 and 2021 can be found in Table 1. The number of FEP patients in a study ranged from 15 to 282 (with a total sum of 2,572 patients). Nine studies included a control group, and one study compared FEP with a non-FEP group. While all the studies were longitudinal, the time period varied from a minimum of 2 months to a maximum of 5 years after the first evaluation. Specifically, a total of 15 studies were carried out over a time period of less than or equal to 1 year, 9 of them covered a following of between 2 and 3 years, and only four were able to follow the patients for 4–5 years. In general, the studies applied scales among the usual ones well-known in neuropsychology, although not necessarily the same, with which the variability and lack of homogeneity of the measurements was observed as a difficulty when establishing general conclusions of the present review (see Table 2). Almost all the studies used correct statistical measures to assess the predictive value of their data (different regression models, hierarchical clustering, or mediation analyses) and, when a control group was used, the authors were concerned with establishing adequate comparability (using matched controls). The reader interested in the specific measures used by each study can consult it in Table 1.

The cognitive variables evaluated in the different articles have been grouped for a better global organization and understanding. These groups are, firstly, a general listing regarding overall cognitive functioning, including IQ and academic performance; secondly, a listing for specific cognitive domains such as processing speed, attention, working memory, verbal memory, visuospatial processing and reasoning/problem solving; and third and lastly, the general domain of social cognition, with a separated listing for ToM (theory of mind), and metacognition.

### 3.1. General cognitive functioning

#### 3.1.1. IQ and overall means obtained in cognitive batteries

Some studies contemplated general cognitive failures through low IQ (mostly applying the WAIS scales) or global cognitive measures through means extracted from cognitive batteries, and their relation with social functioning and disability of FEP patients. For example, a higher IQ at the beginning of the disease was observed to be a predictor of better social functioning with regards to employment and occupation (22) Furthermore, a study identified three predictive clusters of FEP patients that were characterized by distinct cognitive performances. One cluster fell consistently below the sample mean across all cognitive measures, according to the view that there is a subgroup in FEP that has significant globalized cognitive impairments, and premorbid IQ was a significant predictor of group membership. Moreover, the two lower performing clusters both had fewer years of education and were less likely to be employed compared with the subgroup with intact cognition. Poorer general cognitive functioning was associated with poorer participation and functioning in educational, occupational, and social roles at the 6-months follow-up (26). A recent study with the same approaching of clusters observed very similar results, with a trend for significance in the association of social functioning and cognition at the follow-up (47). In addition, another recent study (46) designed cognitive reserve as the sum of IQ plus occupational attainment, and found it significantly related to functionality (though mediated by social cognition as commented further in next sections). In another study, an overall low mean in a battery of neuropsychological tests, indicating worse general cognitive function, predicted higher functional disability after 1 year (but not after 3 years of follow-up) (42). On the other hand, two studies did not find that general cognition was statistically related to social functioning in FEP. One of them (33) reported that quality of life and social functioning were mostly independent of cognitive impairments in a sample of FEP young patients, and the other found no correlation between neurocognition performance and social functioning in a sample of 26 FEP patients followed for 5 years (44).

#### 3.1.2. Academic performance

Education, measured as years of academic achievement, has been suggested to contribute to cognitive reserve, which may act as a protective factor against the deleterious effects of psychotic illness. For instance, although no relationship was detected between cognitive function and a social functioning score (SOFAS) at 5 years in a study, robust correlations appeared between education at onset, premorbid academic adjustment, and overall IQ with full-time occupation and use of disability pension at 5 years (38). The authors suggested that a global measure of intellectual functioning may be a more consistent predictor of functional outcomes than specific indices. As well, they underlined the importance of examining past educational achievement as a possible confound of the relationship between cognitive performance and later functioning. Another study (25) expanded on this idea, directly regarding more years of education at baseline as a predictor for better social and role functioning at 2 years’ follow-up, in a model which, with addition of the attention/vigilance cognitive domain, explained nearly 48% of the variance in social functioning. Indeed, years of education were found to be significant predictors of functional disability at 3-years of disease evolution in another study (42), and were also found to be predictive of role functioning in the follow-up over 4 years in another study (43).

### 3.2. Classic domains of neurocognition

Secondly, all articles which highlighted any of the classic domains of neurocognition and their conclusions were grouped as follows.

#### 3.2.1. Processing speed

Some studies covered the processing speed domain to test its effect on social function in FEP. One study demonstrated that worse processing speed performance at baseline, measured through the WAIS-III Digit Symbol-Coding test, predicted a worse social functioning 1 year later in young FEP patients with adolescent onset psychosis (23), and another study, using the same measure, found that worse processing speed was a significant predictor for real-world functional disability at one-year follow-up (but not at three) in FEP patients, with an OR of 1.57 (42). Finally, a third study, measuring worse processing speed at baseline with the same test, found it to be a significant predictor for most functional outcomes (including social functioning) at 2 years follow-up in FEP patients who developed a schizophrenia syndrome (but not for a group of non-schizophrenia FEP patients) (45). The authors concluded that the performance on processing speed seemed to be a key factor in more severe syndromes, but, however, only a small proportion of the variance was explained by the model, so there must be many other factors that have to be considered. On the other hand, in another study, psychomotor speed was evaluated through a composite score (adding finger tapping and Trail-Making Test) and it was also found highly predictive for poor social functioning (OR = 3.37) after 5 years, even adjusted for antipsychotic drug usage (40). In addition, processing speed, being measured through verbal fluency, predicted recovery (defined as the combination of both symptomatic and social functioning remission) in another study. The authors argued that verbal fluency can be considered to be part of the speed of processing domain, and they concluded that such impairment is a core cognitive deficit in schizophrenia and that from a theoretical perspective, speed of processing and its cognitive components underlie performance in other cognitive domains, being essential for learning and executive functions (39).

#### 3.2.2. Attention

Some studies dealt with attentional variables and concluded that the dysfunction in a patient’s attention, especially sustained attention or vigilance, is predictive for worse social functioning (24, 25, 32, 33, 43). As commented before, the sum of the attention/vigilance domain together with more years of education at baseline formed a predictive model which explained a high proportion (48%) of the variance (25). Interestingly, one study pointed that sustained attention was a good predictor for social functioning, but the prediction was accurate (97%) mainly for the disabled FEP patients but not for the recovered group. The authors emphasized the difficulty of predicting functional recovery and the need to address the role of other factors not commonly explored, like psychosocial interventions or personality, in the functional recovery from FEP (32). Finally, a study found selective attention scores significantly correlated with some aspects of social functioning at baseline and a follow-up of 1 year in FEP patients, though this study did not perform an adequate statistical analysis to address attention as a predictive factor (33).

#### 3.2.3. Working memory

Working memory was addressed in various studies in relation to social function in FEP. One study found that baseline working memory significantly predicted social function at 6-months follow-up (24), however, the same cohort of FEP patients did not show such relationship when evaluated at 2-years (25). In addition, working memory also predicted recovery (defined as the combination of both symptomatic and social functioning remission) in a study (39) while another study (43) went a step further, specifying dysfunction of verbal working memory predicted social and role model dysfunction, being the gain in social functioning over time significantly lower for the low working memory group compared to the high working memory group. Finally, a study found that working memory (24%) and visual learning (6%) predicted a 30% of the variance of functional capacity (defining functional capacity as the abilities needed to perform everyday tasks that are considered necessary for independent functioning in the community) when measured at 10-months follow-up from a FEP (35).

#### 3.2.4. Verbal memory

Three studies examined verbal declarative memory and learning related to a variety of aspects of social functioning, and the relationship was proven to exist, but in general it was not much strong, or it was mediated by another factor. For instance, a recent study (28) found those with poorer verbal memory seem to struggle to maintain performance in roles such as work or education at one-year follow-up; thus, a better verbal memory was a significant predictor for less likelihood to have a poor role functioning. However, a post-hoc mediating analyses confirmed that negative symptoms significantly mediated the relationship between baseline verbal memory and role functioning at follow-up, and the study concluded that cognition may play a subordinate role in predicting functional outcome in early psychosis, compared to chronic. Another study applied a path analysis and demonstrated that verbal memory was indeed related to functioning, but social cognition acted as a mediator between verbal memory and functioning at follow-up (not at baseline), thus concluding that neurocognition is related to functioning, and that social cognition plays a mediating role (46). Another study found verbal memory was a significant predictor on functionality, but explained only 9% of the variance. Adding consecutive months in symptom remission explained 30%, so, they concluded that sustained remission of symptoms, especially of negative symptoms, made a larger contribution to functional outcome than verbal memory. Interestingly, when the analyses were repeated with global cognition replacing verbal memory, a greater effect of global cognition was not found, suggesting that verbal memory may be a stronger predictor of functioning than overall cognitive performance (41). Finally, a study found that verbal memory worsens at 5-years follow-up, but was unrelated to social functioning (44).

#### 3.2.5. Visuospatial processing

This domain was analyzed in three studies, which measured the ability of the individual with FEP to process visual and spatial stimuli (such as social gestures). Visuospatial processing performance at baseline was seen to be a significant predictor for poorer functional outcome after 2 years for a group of FEP patients who did not develop schizophrenia but other psychotic syndromes (bipolar disorder, delusional disorder and brief psychosis) (45). One study tested the hypothesis that baseline cognitive deficits are a stronger predictor of psychosocial functioning than psychotic symptoms. The hypothesis was partially confirmed through the relation between the areas of vocational/academic performance and social relationships, for both of which visual learning was the strongest predictor for functionality after a year (36). However, another study which tested a similar proposal found visuospatial processing not to be a significant predictor for global functioning outcome (28).

#### 3.2.6. Reasoning/problem solving

Some studies examined this cognitive domain, as it is included in the MATRICS Consensus Cognitive Battery. One study (43) observed that the style of reasoning and problem-solving seem to have a bearing on the fulfillment of roles in society, cause role functioning was significantly predicted by a combination of attention, working memory, and reasoning/problem solving.

### 3.3. Social cognition

#### 3.3.1. Social cognition

Social Cognition was assessed in a variety of studies. A social cognition impairment sub-score (from a pooled measure which combined a sub-score of classic cognitive domains and a sub-score of social cognition) was a significant predictor of cognitive change and depression at baseline in one study (29) but unfortunately the relationship was not tested for the follow-up. The authors stated that they failed to find relevant associations between functionality (through the SOFAS) and the combined scale they used to measure social cognition, and suggested that patient judgments regarding their socio-cognitive ability have a minimal correlation to their functional outcomes as rated by mental health professionals (29). Another study, using a panel design with cross-lagged correlations, which allow predictive associations between variables over time while controlling for effects at an earlier time point, found that social cognition at baseline and follow-up assessments robustly and broadly predicted functioning at the 12 month of follow-up across the domains of work, independent living, and social networks (34). Moreover, as commented above, a recent study found that social cognition acts as a mediator between cognitive reserve and global functioning, and also between verbal memory and global functioning (46). However, in another study (30) there were no longitudinal associations between social cognition at baseline and any measure of social functioning at 6-month follow-up. These results held both with and without corrections for multiple comparisons.

#### 3.3.2. Theory of mind

Theory of mind was analyzed to test whether the inference and attribution of mental states of other individuals relates with social functioning. A study (37) observed compromised ToM abilities present even in remitted FEP patients (in a trait-like manner) and, when analyzing longitudinal data, the mediation analyses indicated that neurocognition was a significant mediator in the relationship between two ToM variables (intentionality and appropriateness) and role functioning at 6 months (interestingly, that was their *alternative* mediating model, being their original hypothesis that ToM would be *the* mediator between neurocognition and social functioning, which yielded only a trend for significance). This finding contrasts with another recent study (31), which showed that the addition of the ToM in the STEP3 of a hierarchical regression model of neurocognition on some aspects of social cognition (independent living skills and family) was associated with a significant increase in the variance explained by the model. The authors concluded that theory of mind predicts social functioning better than neurocognition in the case of certain variables. In another study (27) level of social functioning was associated with ToM when age and gender were controlled for, and this association remained significant after controlling for general cognition. This study had a very short time period of following, and they also added that ToM performance was heterogeneous in the whole FEP group, with a large portion of the FEP group showing no deficits. On the other hand, from studies commented previously in the text, one of them demonstrated at the 12-month follow-up that ToM and emotional processing were related to better real-world functioning of FEP patients in a causal manner (34), and another study showed that ToM was also a predictor for problems in social relationships at the 12-month follow-up (36). Nevertheless, we also found contrasting results among the reviewed data: A study found that deficits in ToM were stable over a year but failed to significantly predict social functioning (48), and another study (28) found that some aspects of ToM (attribution and emotion perception) neither were significant predictors for social functioning at 12-months follow-up.

#### 3.3.3. Metacognition

One longitudinal study focused on metacognition in FEP (49), to conclude that a greater metacognitive capacity significantly predicts increased social recovery.

## 4. Discussion

In our review of the recent literature, we have observed that cognition is indeed a key factor in understanding social dysfunction in a first psychotic episode, with significant results reported by 23 of the 28 studies included in our assessment. Anterior reviews, focused in cross-sectional results in patients with schizophrenia, pointed similar conclusions; that is, cognition (neurocognition and social cognition) is significantly related to social functioning in real-world in psychotic patients (50). Our results are in line with this general observation, and extend it to FEP patients who were followed for a variable amount of time and tested with adequate statistical models to find whether cognitive dysfunction at baseline could be predictive for a worse functional outcome at the follow-up. Therefore, the observed initial cognitive deterioration may be, at least in part, the cause of the social functioning deficit. In addition, the anterior reviews observed the variance explained by the statistical model tested in the studies reviewed was generally low. Our results are in line with such observation, which add to the notion of, even being important, cognition function is not enough to explain the level of social disfunction observed in this disease.

A detailed inspection of our review indicated that problems in both overall cognitive ability and IQ at baseline are related to poorer social functioning in the follow-up (22, 26, 38, 42, 43, 46, 47), as we expected based on an initial assessment of the literature. In general, these studies gave support to the notion of cognitive heterogeneity within psychosis patients and confirmed the presence of a part of individuals with psychosis and intact cognitive function, being this classification more based on degree (quantity) of cognitive impairment rather than on specific patterns (quality) of performances in cognitive domains (47). Two of the studies we reviewed did not find any differences (33, 44). In the case of (44) it was concluded that the scale employed to detect social functioning (LSP-J) may not have been sensitive enough for this purpose and their study sample was too small. The (33) study argued that, although they did not detect relationships between greater general cognitive impairment and social functioning, this could be due to the mediating role of social cognition, which was not measured in their study. Moreover, both studies did not address the predictive hypothesis through optimal statistical methodology. With respect to academic performance, all the relevant studies agreed that premorbid academic adjustment improved with a longer academic history, and highlighted a significant positive relationship between the number of years of basic study and social functioning in the longitudinal analyses (25, 38, 42, 43). This indicates that a good academic development during school years can become a protective factor, not so much against the disease itself, but against the loss of social function concomitant to the disease. These results are in line with what we expected based on our assessment of the literature.

On the other hand, when focusing on specific domains of neurocognition, processing speed was found to be a significant predictor of various aspects of social functioning in a bunch of studies (23, 39, 40, 42, 45), and it was even suggested to be essential for the recovery of the patients (39). Attention, specially sustained attention or vigilance (24, 25, 32) was seen significantly predictive of remission (24, 25, 43) and social functioning (24, 25, 32), while selective attention was related to social functioning (33). In the case of working memory, it was seen indeed to be relevant for the prediction of social function: almost all the studies that included this variable (24, 25, 35, 39, 43) agreed that a deteriorated working memory hinders good social function in the follow-up. Interestingly, one cohort of patients show such relation at the 6-moths follow-up (24), but not at the 2 years follow-up (25), thus suggesting that early interventions are useful and capable of rehabilitate both neurocognition and social functioning. Moreover, one study of the above (43) specified that the verbal type of working memory was the most statistically significant. This increased the value to the results observed for the verbal declarative memory domain: various studies (28, 41, 46) observed verbal memory as a significant predictor of poor social functioning. Despite this, one of them found the result mediated by negative symptomatology (28), another one indicated that the variance explained by verbal memory was low (41), and the last one identified social cognition as a mediator between verbal memory and functioning (46). An additional study found that verbal memory worsens at 5 years, but was unrelated to social functioning (44). In sum, it would be of relevance to further investigate this potential relationship between verbal memory failure and social function, since the impaired memory for dynamic social interactions has been suggested as a sign of disease chronification (51), but the results of the longitudinal studies are not as robust as we could expect. On the other hand, the cognitive domain of visuospatial processing did not reach significance as a predictor for social functioning in FEP patients (28) or it was seen as a predictor of functionality mainly for those FEP patients who later developed a non-schizophrenic syndrome (45). Anyway, visual learning (a dominion closer to memory than to visuospatial processing) do was a stronger predictor for functionality after a year (36). In addition to the anterior, role functioning was significantly predicted by a combination of attention, working memory, and the cognitive domain of reasoning/problem solving (43). The general conclusion which emerged from the above-reviewed data is in line with those of previous cross-sectional reviews (52, 53), which showed a significant decline in processing speed, sustained attention, working memory and reasoning/problem related to struggles in social life among individuals with FEP. The present review extends their conclusion to a longitudinal point of view, pointing such cognitive domains are the best predictors for social functioning.

Regarding the studies dealing with social cognition, being this the set of cognitive processes involved in social situations, it makes sense that, if this ability is impaired, it will be an important cause of social functioning deficit in an individual with psychosis. This point has been partially consistent with the results of our review. A study (34) applied an optimal statistical analysis for testing causality, and observed that baseline social cognition impairment led to worse real-world functioning in a 12-month follow-up; however, another two studies (29, 30) did not find such a relationship, one of them find some cross-sectional associations but it did not test for in the follow-up (29), and the other (30) did not find significance for the longitudinal associations. In addition, some authors have suggested that social cognition would play a mediating role in between neurocognition and outcomes of social functionality, which would underline its importance from a clinical point of view, especially when detecting a first episode and undertaking early intervention (50). Specifically, a study (37) identified social cognition acting as a mediator (through the Sobel test) between cognitive reserve and functioning, and between verbal memory and functioning, both of them at follow-up (not at baseline). In another study, centered only in the ToM, the authors tested the hypothesis of social cognition as a mediator between neurocognition and social functioning, yielding a trend for significance (46). Regarding the rest of articles which measured the influence of ToM (as a specific part of social cognition) in functioning, they did not unanimously point to a relationship with social functioning, which we did not expect, since inference of the mental state of the people with whom we interact should be a key factor in social functioning. In fact, while some studies observed ToM significantly predicted social functioning (27, 34, 36) some other did not find such relation (28, 48), or found a poor relation with low variance explained (31), or saw the relationship of ToM on role functioning significantly mediated by neurocognition (37). In the case of the studies with non-significant results (28, 48), both agreed that their measurement of ToM was influenced by negative symptoms. In the first, negative symptoms significantly mediated between baseline memory and role functioning at follow-up (28). Moreover, the severity in social cognition impairment (which was a predictor of cognitive change and depression) appeared to be specifically associated with negative symptoms in another study (29). Therefore, it seems to be that negative symptoms can act as a mediator between neurocognition, social cognition, and social functioning (48). These findings need further investigation since symptoms often improve, whereas social functioning does not. A study (41) proposed that cognitive abilities improve following early remission of symptoms, both positive and negative, and that this remission, in turn, correlates with an improvement in social function; thus, symptomatic remission is undoubtedly important and impacts on the social function of the individual in a parallel way to cognitive function and may even influence it. Finally, in regards to metacognition, one study showed it was a significant predictor of change in functional outcome from baseline to follow-up, with a high proportion of variance explained and independently of negative symptoms (49). Metacognitive capacity has been suggested to mediate the relationship between neurocognition and the individual’s social and occupational function in cross-sectional studies (7). This suggests that the ability to think about own’s thinking, specifically, the mental processes involved in this from a clinical point of view, could be a target for rehabilitation and early intervention in social functioning. Metacognition therapies that focus on maintaining optimal social function may be one of the most appropriate steps towards addressing social rehabilitation in FEP.

## 5. Conclusion

We have repeatedly observed that cognitive deficits impact on the social functioning of the FEP patient from the beginning of the disease and across the time and, therefore, the principal clinical implication of this review is that the rehabilitation interventions must take this factor into account. The neurocognitive domains most closely related to social functioning in FEP seem to be processing speed, sustained attention and working memory, but a general overall cognitive disfunction or a low IQ are also very important for an adaptative social functioning in the future. The initial assessment of the FEP patient should take these aspects into account, as well as consider the patient’s academic trajectory. Regarding social cognition and its effect on social functioning, the findings were not unanimous. A significant predictive relationship was found in some studies, but we found also quite studies with little or no relationship, both in terms of social cognition in general and specifically ToM or metacognition. It is interesting to add that several of the articles found a complex relationship between social cognition, neurocognition, social functioning and negative symptoms, pointing social cognition as a modulator of neurocognition but being modulated as well by negative symptoms. In any case, given the results of the review and in the absence of more studies that clearly establish the kind of mediation relationship which seems to exist among these variables, we conclude that an initial evaluation of social cognition in a FEP patient should be essential. Finally, the present review has a number of strengths and weaknesses. As strengths, longitudinal studies are exhaustively presented, with adequate statistical power and using samples of FEP patients. As weaknesses, we highlight the possible selection bias of the studies, both due to the inclusion criteria and to the articles that are usually reported in the literature, with a greater tendency to show significant results. In addition, the different studies were not observed to be homogeneous when choosing the scales to assess the different constructs, and furthermore, the theoretical constructs themselves are far from being unified in the literature (especially with regard to those that have to do with social cognition) which limits the general conclusions that can be drawn.

## Data availability statement

The original contributions presented in the study are included in the article/Supplementary material, further inquiries can be directed to the corresponding author.

## Author contributions

MM-F, MG, and JS contributed to conception and design of the systematic review, and to the definite selection of the included articles. MM-F organized the database and Table 1, and wrote the first draft of the manuscript. MG and JS revised and corrected the manuscript and Tables 1, 2. All authors contributed to final revision and approved the submitted version.

## Funding

This study was supported by grants from the Generalitat Valenciana (PROMETEO/2020/024; FIS PI20/00473).

## Conflict of interest

The authors declare that the research was conducted in the absence of any commercial or financial relationships that could be construed as a potential conflict of interest.

## Publisher’s note

All claims expressed in this article are solely those of the authors and do not necessarily represent those of their affiliated organizations, or those of the publisher, the editors and the reviewers. Any product that may be evaluated in this article, or claim that may be made by its manufacturer, is not guaranteed or endorsed by the publisher.
